# Non-clinical Safety Evaluation of Camelina Oil: Acute and 12-Week Oral Toxicities

**DOI:** 10.5812/ijpr-140666

**Published:** 2024-04-08

**Authors:** Kambiz Varmira, Danial Kahrizi, Azarm Sanjari, Khodabakhsh Rashidi, Leila Hosseinzadeh, Niloufar Amin, Fereshteh Jalilian

**Affiliations:** 1 Research Center of Oils and Fats, Health Technology Institute, Kermanshah University of Medical Sciences, Kermanshah, Iran; 2 Department of Agricultural Biotechnology, Faculty of Agriculture, Tarbiat Modares University, Tehran, Iran; 3 Jahad Daneshghi Univesity, Kermanshah, lran; 4 Pharmaceutical Sciences Research Center, Health Institute, Kermanshah University of Medical Sciences, Kermanshah, Iran

**Keywords:** Camelina Sativa, Alanine Aminotransferase, Acute Toxicity, Sub-chronic Toxicity, Biochemical Profile, Liver Histology

## Abstract

This study assessed the acute and sub-chronic toxicity of Camelina oil, a well-known oil rich in polyunsaturated fatty acids that enhance cellular immunity and human health, in Wistar rats. Wistar rats, 5 per sex per group, were randomly assigned to three groups for acute (14 days) toxicity studies and five groups for sub-chronic (90 days) toxicity studies. In the acute study, Camelina sativa oil was administered orally at a single dose of 5000 mg/kg of body weight (BW). The positive control group received a single dose of 5 000 mg/kg BW Canola oil by gavage. In the sub-chronic study, Groups III-V received 250, 500, and 1 000 mg/kg BW of Camelina oil, while Groups I and II received ultra-pure water and Canola oil at a dose of 500 mg/kg BW, respectively. Throughout the experiment, clinical signs, mortality, and body weight were monitored. At the end of the sub-chronic study, hematological, biochemical, and histopathological investigations were conducted. Administration of Camelina oil and Canola had no significant effect on daily weight gain (P > 0.05) of the test rats. Serum calcium levels decreased while phosphorous levels increased in male rats treated with Camelina oil. Other hematological and biochemical parameters showed no significant differences or dose-response effects between control and seed oil groups in both sexes (P < 0.05). Moreover, in animal necropsy, there were no apparent lesions in the liver, heart, and kidney organs in any of the doses administered. In conclusion, the results suggest that oral administration of Camelina oil is unlikely to be toxic. Therefore, the possibility for the development of future human nutrition should be considered.

## 1. Background

Nowadays, there is growing interest in using vegetable oils as a dietary supplement due to their potential positive impact on animal and human health (1), particularly regarding their high content of polyunsaturated fatty acids (2). Of particular note are the omega-3 long-chain polyunsaturated fatty acids (LC-PUFAs), including eicosapentaenoic acid (EPA) and docosahexaenoic acid (DHA), which have been shown to promote human health (3), fetal growth, cardiovascular health (4), as well as immune and anti-inflammatory responses.

Until recently, vegetable oils and fish oils have provided the primary sources of EPA and DHA for human consumption, but due to sustainability and accessibility concerns related to fish oils, more efforts have been made towards scaled production of oilseed-crop-based sources to increase the availability of EPA and DHA (5-7).

Additionally, oils rich in α-Linolenic Acid (ALA) have gained attention due to the expected cardio-protective health benefits associated with the fatty acid (8). α-Linolenic Acid is the metabolic precursor of the LC-PUFA that is converted to EPA and DHA; however, this conversion happens at low rates (5).

Camelina sativa, an oil plant, is among the richest sources of ALA omega-3 fatty acid and natural antioxidants such as tocopherols (8-10). These features make Camelina oil a potential alternative to other plant-based food-grade oil products. Camelina sativa oilseed also contains stearidonic acid, an intermediate in the biosynthesis of eicosapentaenoic acid from alpha-linolenic acid, which can be effective as a dietary supplement to improve the status of LC-PUFAs in consumers (5).

However, the potential health benefits of Camelina sativa are overshadowed by concerns over high dietary exposure to two anti-nutrient compounds: Erucic acid and Glucosinolate (11). The FDA has determined that the maximum allowed limit of Erucic acid in edible oils should be less than 2%. Exposure to high levels of Erucic acid is associated with myocardial lipidosis and heart lesions which can adversely affect the liver or heart tissues (12). Glucosinolate may also have some adverse effects on thyroid function including enlargement of the thyroid gland (13).

## 2. Objectives

Hence, it becomes essential to assess the safety of Camelina oil in order to establish its safety for longer periods of consumption. Therefore, to provide data for establishing a safe dosage for clinical application, the current study was carried out to evaluate the toxicity effect of Camelina oil on the histology of vital organs and biochemical parameters, following long-term administration (90 days) in Wistar rats.

## 3. Methods

### 3.1. Animal Experiments

Wistar rats, 8 weeks old, with average weights of (243 ± 25 g) for males and (202 ± 17 g) for females, and (215 ± 24 g) for males and (222 ± 13 g) for females were used. The animals were maintained in the animal breeding unit of the Pharmacy Faculty, Kermanshah University of Medical Sciences (Kermanshah, Iran). Prior to the commencement of experiments, these animals were housed in the laboratory in stainless steel wire cages under laboratory conditions of 12-hour light-dark cycles with 2 weeks’ acclimatization, while the temperature (23 ± 2°C) and humidity were kept constant. The rats were fed a conventional laboratory diet and provided with tap water without any limitation, following overnight fasting before experimentation. The animals were first numbered and then randomly assigned into groups.

The experiments were approved by the Research Ethics Committee of Kermanshah University of Medical Sciences with the ethical code: IR.KUMS.REC.1397.556. The protocol of the study was in accordance with the Helsinki Declaration.

### 3.2. Plant Collection and Extracts Preparation

Fresh Camelina and Canola oilseeds were collected from the Biston Shafa Company in Kermanshah, Iran in 2016. In this research, the “Soheil” cultivar of Camelina sativa plant has been used, which was released by Danial Kahrizi (one of the authors of this article) and has been utilized in many research studies (reference). Kahrizi, D. (2018). Report introducing the Soheil cultivar of Camelina Sativa plant for cultivation in different parts of the country. Seed and seedlings certification. Summer. 6(2): 24 - 27. The oil extraction from each of the oilseeds was carried out using a cold press machine available in the laboratory of pant production and genetics, faculty of agriculture, Razi University, Iran. The extracted oil was filtered with Whatman filter paper, collected, and stored in a sealed brown bottle at 4°C for further analysis.

### 3.3. Determination of Fatty Acid Composition

Esterified fatty acids were determined after transesterification according to the AOCS Official Method Ce 1h - 05. A Hewlett–Packard model 5890 II gas chromatograph (Agilent Technologies, Avondale, PA, USA) with a flame ionization detector equipped with a Supelcowax 10 column (30 m × 0.25 mm × 0.25 µm) (SUPELCOWAX ™ 10, Millipore Sigma™, USA) was used. The experimental conditions were regulated as follows: The injection ratio was 1:25, hydrogen flow was maintained at 1 mL/min, oven temperature was set at 220 ± 1°C, and detector temperature at 240°C. The column temperature was programmed from an initial temperature of -20°C (for 5 minutes) to a final temperature of 280°C (for 5 minutes). The temperature increased from -20°C to 30°C at a rate of 10°C/minute, from 30°C to 200°C at a rate of 5°C/minute, and finally from 200°C to 280°C at a rate of 10°C/minute. The oil content of the seed samples was calculated and reported based on their dry weight percentage, and the amount of fatty acids was determined based on the total oil percentage by comparing their subpeak area with standard samples (C: 12 - C: 24, Sigma Company). The identification of the fatty acids was conducted using the National Institute of Standards and Technology (NIST) Mass spectral library by Nazgol Company with test method 2 – 1 - 13126 (14).

### 3.4. Acute Oral Toxicity Study of Camelina Oil

Thirty rats were randomly assigned to two groups, each consisting of five males and five females.

• Group 1: Normal Control (NC): These rats were administered only with the equivalent volume of ultrapure water as used for the administration of plant seed oil.

• Group 2: Treatment Rats were administered a single dose of Camelina seed oil, 5 000 mg/kg (OECD, 2008), via the gavage method, which is chosen as a general dose in a single-dose toxicity study. The dose of extract oil was diluted with ultrapure water to a constant volume of 2 mL.

General clinical observations such as any signs of toxicity (convulsions, drowsiness, changes in animal weight, and mortality) were made at specific daily times for 14 days.

### 3.5. Sub-chronic Toxicity Studies

For sub-chronic toxicity studies, fifty Wistar rats were randomly allocated to five groups of 10 rats each (five males and five females) and orally treated once daily for 90 days via the gavage method. Sub-chronic toxicity of Camelina sativa was evaluated in accordance with Organization for Economic Co-operation and Development (OECD) guidelines (OECD, 1998). Rats in group I (control) received only 2.0 mL ultrapure water. Rats in group 2 were orally administered a daily single dose of 500 mg/kg BW of Canola oil, considered as a positive control group. The rats in groups 3, 4, and 5 received daily single doses of 250, 500, and 1000 mg/kg BW of Camelina oil, via gavage, respectively.

Throughout the treatment period, all rats were also observed daily for body weight, severe toxicity, mortality, or any other changes. During the weight measurements, alterations in the behaviors, skin, and physical appearance of the animals were monitored.

### 3.6. Rat Blood Sampling

At the end of the treatment period, rats were subjected to 12 - hour fasting with free access to water before blood sampling on day 90. The rats were anesthetized, and blood samples were withdrawn intracardially using a 5 mL syringe and collected in 5 mL sample bottles containing heparin for an hour. Then, 3 mL of blood separately was emptied into a vial for hematological evaluation, and an additional 2 mL was stored until required for measurement of biochemical parameters.

### 3.7. Hematological and Biochemical Analysis

To obtain the serum, the blood samples were centrifuged for 15 minutes at 1 500 g using a serum separation reagent. Afterward, the serum was analyzed using a Sysmex K1000 fully automated hematology analyzer and COBAS Mira S chemistry analyzer (Roche Diagnostic Systems, West Sussex, England). The hematological parameters to determine included: Red blood cell (RBC) count, white blood cell (WBC) count, hemoglobin (Hb) concentration, platelets, mean corpuscular volume (MCV), mean corpuscular hemoglobin (MCH), and mean corpuscular hemoglobin concentration (MCHC).

Biochemical parameters include: Blood sugar, total cholesterol, triglycerides, total bilirubin (Bit), direct bilirubin (Bid), albumin, enzyme activities of aspartate aminotransferase (AST), alanine aminotransferase (ALT), alkaline phosphatase (ALP), urea, creatinine, calcium, sodium, chloride, potassium were determined to investigate hepatic and renal functions, respectively.

### 3.8. Necropsy and Histopathology

At the end of the trial and after sacrificing control and treatment groups, in order to investigate the tissues microscopically, fragments of different organs including the heart, liver, kidney, spleen, and lung of rats were carefully excised, fixed in 10% neutral buffered formalin, and embedded in paraffin blocks. The embedded tissues were sectioned, stained with hematoxylin and eosin, and examined under the light microscope to further investigate any physical appearance or histopathological changes. The weights of the livers, hearts, and kidneys were measured by an electric balance sensitive to 0.0001 g. The relative organ weight was calculated for all the rats by dividing the absolute organ weight by the final rat weight and multiplying by one hundred.

### 3.9. Statistical Analysis

In the present study, all the measured parameters are reported as the mean and standard deviation (mean ± SD). The difference between treatment and control groups was separately determined for males and females using one-way analysis of variance (ANOVA) followed by Tukey’s multiple comparison test. Differences in means were considered significant at P < 0.05 and not significant at P > 0.05. All statistical analyses were performed using GraphPad Prism®, version.

## 4. Results

### 4.1. Fatty Acid Composition

As presented in Table 1, Camelina oil contained 48.63% polyunsaturated fatty acids, with C18: 3 possessing the highest value at 29.56%. These results were consistent with previous studies (15, 16). The content of C18: 2 in Camelina oil was 19.07%. Raczyk et al. reported a highly similar amount for linoleic fatty acid (between 18 and 19%) (16). The amount of C18:1 in Camelina oil was found to be 15.19%, slightly lower than the average recorded by Raczyk et al. and Rodriguez-Rodriguez et al. (16.1% and 16.7%, respectively) (16, 17). The tested Camelina oil had a low content (12.04%) of saturated fatty acids (SFA).

**Table 1.** Fatty Acid Profile of Camelina Oil

**Entry**	**Factor/Treatment**	**Common Name**	**Content of Fatty Acid ** ^a^
**1**	C12: 0	Lauric acid	0.04
**2**	C14: 0	Myristic acid	0.01
**3**	C16: 0	Palmitic acid	6.50
**4**	C16: 1	Palmitoleic acid	0.50
**5**	C17: 0	Heptadecanoic acid	0.04
**6**	C17: 1	Cis-10-heptadecenoic	
**Acid**	0.04		
**7**	C18: 0	Stearic acid	2.70
**8**	C18: 1	Oleic acid	15.19
**9**	C18: 2	Linoleic acid	19.07
**10**	C18: 3	γ-Linolenic acid	29.56
**11**	C20: 0	Arachidic acid	2.19
**12**	C20: 1	Eicosenoic acid	14.91
**13**	C22: 0	Behenic acid	0.45
**14**	C22: 1	Erucic acid	1.33
**15**	C22: 2	Cis-13,16 docosadienoic acid	ND
**16**	C24: 0	Lignoceric acid	0.11
**17**	C24: 1	Nervonic acid	0.67
**18**	∑ SFA a	Saturated fatty acids	12.04
**19**	∑ MUFA a	Monounsaturated fatty acids	32.64
**20**	∑ PUFA a	Polyunsaturated fatty acids	48.63

^a^ Values are expressed as percentage (%).

### 4.2. Acute Toxicity Study of Camelina Oil

The data from the acute toxicity investigation over a 14 day period show that Camelina sativa oilseed was relatively safe; no mortality, sudden death, or significant changes were observed in both control and treatment groups. This suggests that the LD50 of Camelina oil exceeded 5 000 mg/kg.

### 4.3. Sub-chronic Toxicity Study of Camelina Sativa Oil

#### 4.3.1. Clinical Observations and Mortality

Clinical observations were performed daily before and after the dosing periods. The results of these records did not show any changes in physical characteristics and exploratory behavior.

There were no deaths recorded throughout the experiment, indicating that the rats tolerated the 90 day oral administration of the oilseed well.

**Figure 1.**
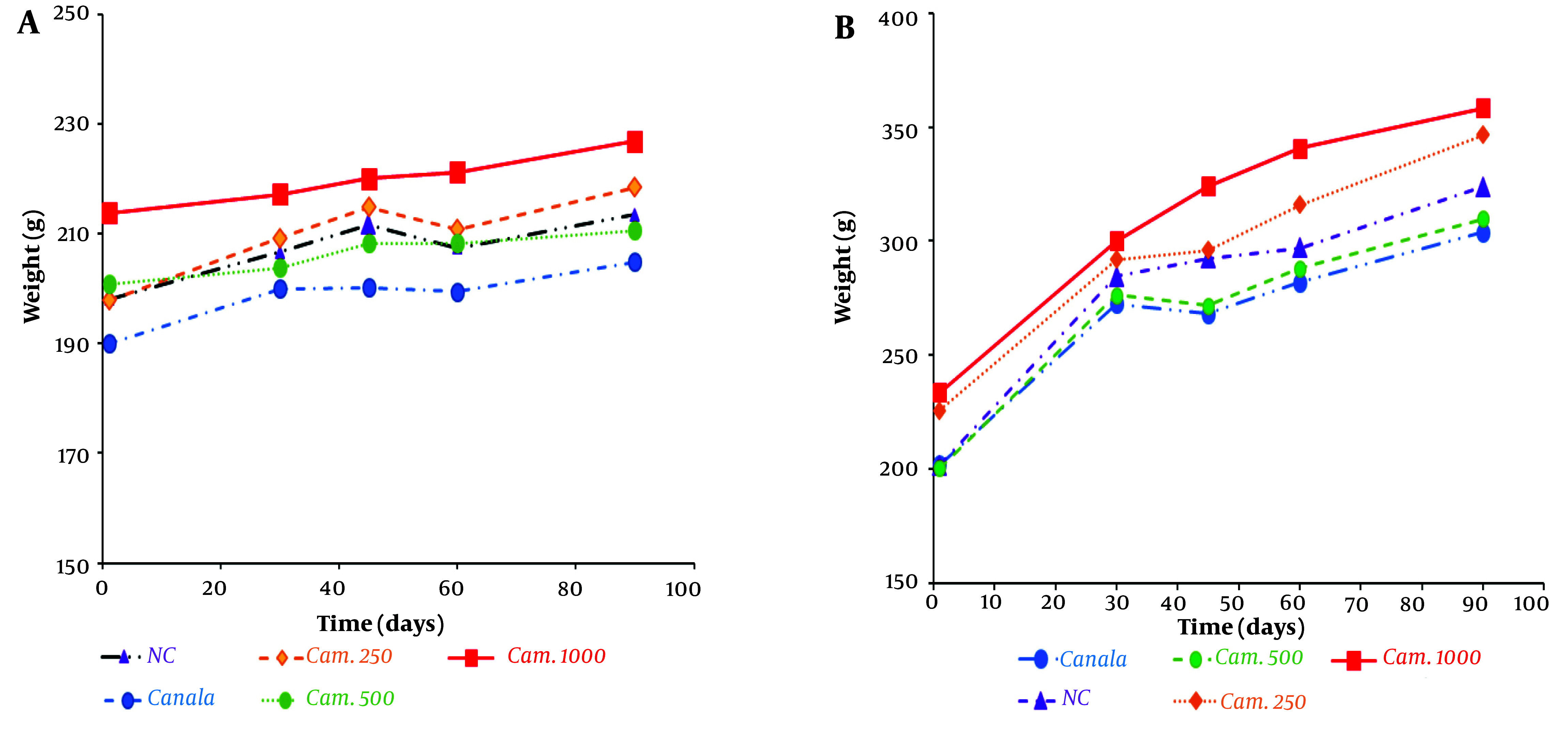
Effect of the plant seed oils on the body weight changes of A, female and B, male Wistar rats after sub-chronic administration

The levels of weight gain in the animals might be sensitive and simple indexes to evaluate the toxicity effect. The data presented in Figure 1 show that there was no significant increase (P > 0.05) in the daily mean body weights of the test animals administered Camelina oil at doses of 250, 500, and 1000 mg/kg, as well as Canola oil at 500 mg/kg BW, relative to the respective controls. However, male rats gained more mean daily weight (P < 0.05) compared to females; alterations in the daily weight gain of male rats administered a high dose of the seed oil (1 000 mg/kg) clearly show an increasing trend, while female weight gain occurred with a slower slope (Figure 1B). 

#### 4.3.2. Hematology and Biochemical Profile

It is evident that there were no significant differences (P > 0.05) in the determined hematological indices (HB, PCV, WBC, RBC, HCT, MCH, PLT, and MCV) among all the treated rats exposed to 90 days of sub-chronic treatment with different doses of Camelina and 500 mg/kg Canola oils (Table 2). 

**Table 2.** Hematological Profile of Wistar Rats Treated with Camelina and Canola Oil ^a^

Sex and Dosemg/kg	WBC (10^3^/µL)	RBC (10^3^/µL)	HGB (g/dL)	HCT (%)	MCV (Fl)	MCH (pg)	MCHC (g/dL)	PLT (10^3^/µL)
**Female**								
Control	3.18 ± 0.45	7.85 ± 0.37	14.22 ± 0.26	41.50 ± 0.96	52.90 ± 2.18	18.14 ± 0.74	34.28 ± 0.30	880.80 ± 68.66
Canola ^b^	4.54 ± 0.58 ^b^	7.27 ± 0.44	13.46 ± 0.72	39.44 ± 1.83	54.34 ± 1.46	18.52 ± 0.29	34.12 ± 0.58	906.60 ± 48.83
250 ^b^	4.44 ± 0.23 ^b^	7.74 ± 0.48	14.00 ± 0.53	41.58 ± 1.07	53.88 ± 2.66	18.46 ± 0.52	33.66 ± 0.67	902.80 ± 50.40
500	3.98 ± 0.48	7.13 ± 0.53	13.62 ± 0.73	40.36 ± 2.71	55.22 ± 1.22	18.60 ± 0.41	33.76 ± 0.80	859.00 ± 63.77
1000	3.84 ± 0.46	7.56 ± 0.37	14.22 ± 0.54	41.66 ± 0.77	55.10 ± 2.26	18.60 ± 0.44	34.04 ± 0.74	867.00 ± 91.80
**Male**								
Control	4.08 ± 0.42	7.55 ± 1.11	13.82 ± 0.86	41.56 ± 1.81	53.36 ± 3.26	18.04 ± 1.12	33.26 ± 1.76	923.60 ± 32.35
Canola	4.16 ± 0.98	8.14 ± 0.30	14.32 ± 0.33	41.34 ± 0.83	50.84 ± 1.21	17.60 ± 0.51	34.64 ± 0.44	873.00 ± 88.38
250	5.42 ± 1.31	8.33 ± 0.38	14.60 ± 0.34	43.06 ± 1.59	51.74 ± 1.93	17.56 ± 0.65	33.86 ± 0.62	832.80 ± 90.53
500	6.14 ± 1.43	8.31 ± 0.26	14.38 ± 0.78	43.10 ± 3.19	52.52 ± 3.39	17.52 ± 0.74	33.42 ± 1.05	908.40 ± 16.95
1000	6.06 ± 1.02	7.94 ± 0.30	14.18 ± 0.44	41.56 ± 1.37	52.36 ± 2.03	17.88 ± 0.69	34.10 ± 0.52	892.00 ± 75.60

Abbreviations: WBC, white blood cell; RBC, red blood cell; MCV, mean corpuscular volume; MCH, mean corpuscular hemoglobin; MCHC, mean corpuscular hemoglobin concentration.

^a^ Results are expressed as mean ± SD.

^b^P < 0.01.

Compared with the control group, WBC levels markedly increased in male rats treated with 250 mg/kg of Camelina oil and 500 mg/kg of Canola extract (P < 0.05). However, there were no significant elevated variations in the WBC values of the Camelina oil-treated male rats (P > 0.05 vs. control).

The biochemical and enzymatic profile corresponding to liver and kidney functions are displayed in Tables 2 - 4. Albumin concentration in female rats was not affected by the administration of Camelina or Canola oil (P < 0.05). The concentration of this parameter in male rats decreased only at the 1 000 mg/kg level compared to the control group (P < 0.01). Administration of Camelina or Canola oils to female rats did not change the concentration of alanine aminotransferase (P > 0.05), but it decreased in males (P < 0.01). Aspartate aminotransferase and alkaline phosphatase were not affected by the administration of edible oils in both sexes (P < 0.05).

**Table 3.** Biochemical Profile of Male and Female Rats Treated with Camelina and Canola Oil ^a^

Sex and Dose (mg/kg)	Blood Sugar (mg/dL)	Triglycerides (mg/dL)	Cholesterol (mg/dL)	Creatinine (mg/dL)	Urea (mg/dL)
**Female**					
Control	83.20 ± 8.53	123.80 ± 5.26	94.60 ± 3.91	0.68 ± 0.09	75.40 ± 2.70
Canola	86.00 ± 11.38	119.40 ± 5.64	93.40 ± 3.91	0.66 ± 0.04	82.20 ± 5.40
250	79.40 ± 6.11	122.00 ± 5.29	88.60 ± 4.83	0.66 ± 0.07	75.20 ± 6.76
500	76.00 ± 11.02	116.80 ± 2.95	90.60 ± 10.97	0.71 ± 0.08	87.60 ± 5.77
1000	74.80 ± 13.33	128.80 ± 5.26	93.60 ± 3.29	0.72 ± 0.05	79.40 ± 12.82
**Male**					
Control	96.40 ± 11.48	65.80 ± 1.48	84.80 ± 12.40	0.67 ± 0.07	72.00 ± 10.56
Canola	85.20 ± 13.05	61.80 ± 4.02	92.60 ± 3.51	0.66 ± 0.02	67.00 ± 3.54
250	78.00 ± 13.51	62.60 ± 4.53	82.40 ± 10.09	0.66 ± 0.05	80.40 ± 6.77
500	83.60 ± 10.95	62.80 ± 2.17	85.20 ± 2.68	0.67 ± 0.03	70.40 ± 6.11
1000	87.20 ± 14.72	72.80 ± 4.44	93.00 ± 1.58	0.68 ± 0.05	70.80 ± 5.26

^a^Results are expressed as mean ± SD.

**Table 4.** Effect of Sub-chronic Administration of Camelina and Canola Oils on Some Biochemical Parameters of Rats

Sex and Dose (mg/Kg)	Albumin (g/dL)	TotalBilir (mg/dL)	DirectBilir (mg/dL)	ALT (U/L)	AST (U/L)	ALP (U/L)
**Female**						
Control	3.58 ± 0.22	1.83 ± 0.33	0.11 ± 0.05	392.40 ± 91.26	95.40 ± 7.30	206.00 ± 39.39
Canola	3.72 ± 0.15	1.93 ± 0.14	0.12 ± 0.04	484.80 ± 76.91	123.20 ± 32.77	349.40 ± 76.39
250	3.540.15	2.04 ± 0.03	0.12 ± 0.01	422.80 ± 90.00	90.00 ± 9.57	286.60 ± 85.49
500	3.62 ± 0.19	2.07 ± 0.09	0.12 ± 0.02	471.00 ± 98.57	118.60 ± 21.88	298.00 ± 87.24
1000	3.78 ± 0.16	2.05 ± 0.10	0.17 ± 0.01	526.00 ± 51.58	147.00 ± 3.54	308.00 ± 59.39
**Male**						
Control	3.94 ± 0.11	1.96 ± 0.03	0.16 ± 0.04	402.80 ± 101.57	75.20 ± 10.83	428.20 ± 26.17
Canola	3.96 ± 0.09	1.92 ± 0.03	0.13 ± 0.05	262.80 ± 28.58 ^a^	70.40 ± 6.91	428.60 ± 41.51
250	3.84 ± 0.24	1.89 ± 0.03	0.13 ± 0.03	266.00 ± 18.51^a^	82.40 ± 14.72	402.00 ± 50.81
500	3.68 ± 0.40	1.87 ± 0.06	0.12 ± 0.02	272.80 ± 31.32 ^a^	77.00 ± 1384	422.00 ± 33.64
1000	3.28 ± 0.13^a^	1.86 ± 0.06	0.16 ± 0.06	323.40 ± 74.58 ^a^	79.00 ± 10.00	409.20 ± 55.89

Abbreviations: AST, aspartate aminotransferase; ALT, alanine aminotransferase; ALP, alkaline phosphatase.

^a^P < 0.01.

The possibility of any significant alteration in the concentration of electrolytes in blood samples of animals, after treatment with the oils, was also examined and tabulated in Table 5. The level of potassium in female rats showed a statistically significant difference after administration of Camelina and Canola oil at the dose of 250 mg/kg and 500 mg/kg, respectively, compared to the control group (P < 0.01), whereas no differences were seen in the case of male rats. The blood calcium and phosphorus levels of male rats administered Camelina or Canola oil recorded a significant reduction in blood calcium (P < 0.05) and a considerable increase in the level of blood phosphorus relative to the control (P < 0.01). Moreover, a significant decrease was observed in the ALT activity in the serum of male rats in all treated groups compared to that of the control (P < 0.05). Among the groups receiving Camelina oil, group I (250 mg/kg) showed a greater significant decrease in enzyme levels.

**Table 5.** Concentration of Minerals in Blood Samples of Wistar Rats After Sub-chronic Study ^a^

Sex and Dose (mg/kg)	Na (mEq/L)	K (mEq/L)	Ca (mg/dL)	P (mg/dL)
**Female**				
Control	138.40 ± 1.34	7.16 ± 0.21	9.46 ± 0.15	8.10 ± 0.12
Canola	138.80 ± 1.10	8.44 ± 0.71 ^b^	9.38 ± 0.08	8.10 ± 0.07
250	138.80 ± 1.79	9.00 ± 0.39 ^b^	9.28 ± 0.18	7.88 ± 0.41
500	139.60 ± 1.67	7.98 ± 0.08	9.24 ± 0.18	8.16 ± 0.15
1000	139.80 ± 0.84	7.10 ± 0.10	9.44 ± 0.09	8.16 ± 0.11
**Male**				
Control	139.80 ± 0.84	7.04 ± 0.11	10.12 ± 0.16	5.24 ± 0.23
Canola	139.40 ± 1.14	7.08 ± 0.08	9.14 ± 0.11 ^b^	8.14 ± 0.11 ^b^
250	139.40 ± 0.89	7.06 ± 0.11	9.28 ± 0.19 ^b^	8.08 ± 0.08 ^b^
500	140.20 ± 1.92	7.06 ± 0.15	9.36 ± 0.19 ^b^	8.24 ± 0.21 ^b^
1000	139.20 ± 0.84	7.40 ± 0.12	9.38 ± 0.13 ^b^	8.10 ± 0.12 ^b^

^a^ Results are expressed as mean ± SD.

^b^ P < 0.01.

#### 4.3.3. Organ Coefficients

The ratios of liver, kidney, heart, spleen, and lung weights to body weight (organ coefficients) of Wistar rats administered with Camelina and Canola oil after completing the sub-chronic study are summarized in Table 6. 

**Table 6.** Relative Organ Weight of Wistar Rats Treated with Camelina and Canola Oils ^a^

Sex and Treatment (mg/kg)	Liver	Kidney	Heart	Spleen	Lung
**Female**					
Control	2.698 ± 0.29	0.350 ± 0.05	0.403 ± 0.05	0.401 ± 0.06	0.650 ± 0.17
Canola	2.766 ± 0.19	0.348 ± 0.03	0.374 ± 0.02	0.356 ± 0.06	0.761 ± 0.08
250	2.744 ± 0.35	0.365 ± 0.03	0.362 ± 0.03	0.374 ± 0.04	0.704 ± 0.03
500	2.700 ± 0.19	0.341 ± 0.03	0.391 ± 0.05	0.405 ± 0.05	0.754 ± 0.12
1000	2.700 ± 0.08	0.354 ± 0.02	0.369 ± 0.04	0.418 ± 0.06	0.756 ± 0.12
**Male**					
Control	2.425 ± 0.11	0.299 ± 0.03	0.374 ± 0.02	0.321 ± 0.06	0.611 ± 0.06
Canola	2.366 ± 0.16	0.312 ± 0.04	0.348 ± 0.02	0.278 ± 0.05	0.545 ± 0.06
250	2.449 ± 0.18	0.309 ± 0.03	0.380 ± 0.03	0.325 ± 0.05	0.549 ± 0.06
500	2.788 ± 0.24 ^b^	0.366 ± 0.03 ^b^	0.401 ± 0.02	0.336 ± 0.02	0.0641 ± 0.09
1000	2.702 ± 0.15	0.309 ± 0.03	0.353 ± 0.04	0.331 ± 0.04	0.527 ± 0.04

^a^ Results are expressed as mean ± SD and (%).

^b^P < 0.05.

The organ coefficients of the liver and left kidney were statistically similar in administered and control female rats (P > 0.05), whereas male rats administered with Camelina oil at 500 mg/kg revealed significant alterations in the weight coefficients of these organs (P < 0.05). The ratios of heart, spleen, and lung to body weight in both male and female rats were not significantly different compared to control rats (P < 0.05).

#### 4.3.4. Histopathology Studies

**Figure 2.**
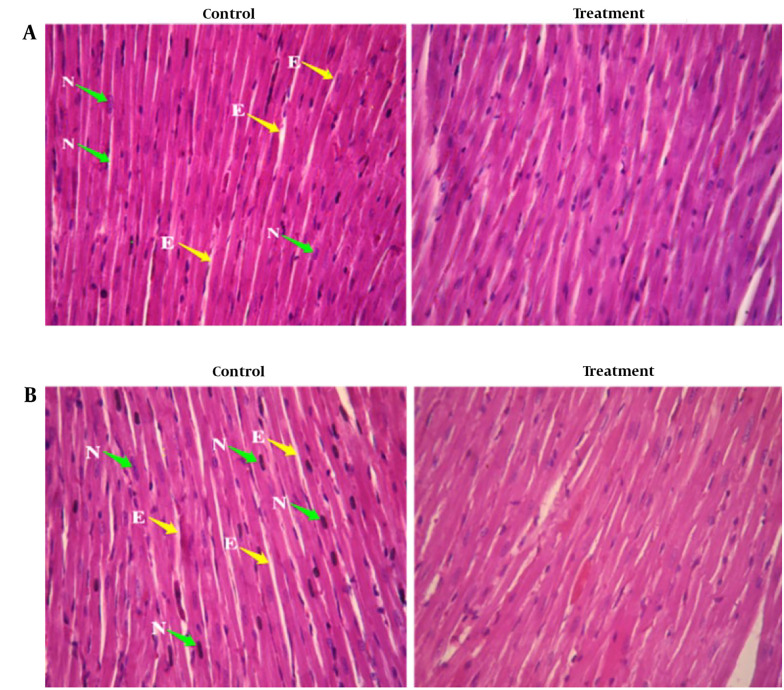
Photomicrograph of the sectioned heart of A, female rats: Control and treated with 1 000 mg/kg Camelina oil; B, male rats: Control and treated with 1 000 mg/kg Camelina oil N: Nuclear, E: Endomysium.

**Figure 3.**
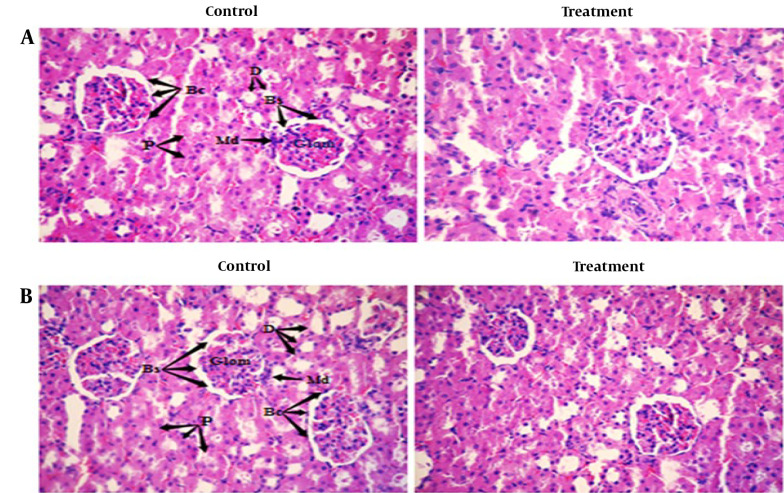
Photomicrograph of sectioned kidney of A, female rats: Control and treated with 1 000 mg/kg Camelina oil; B, male rats: Control and treated with 1 000 mg/kg Camelina oil. Bs: Bowman, Md: Macula densa, D: Distal.

**Figure 4.**
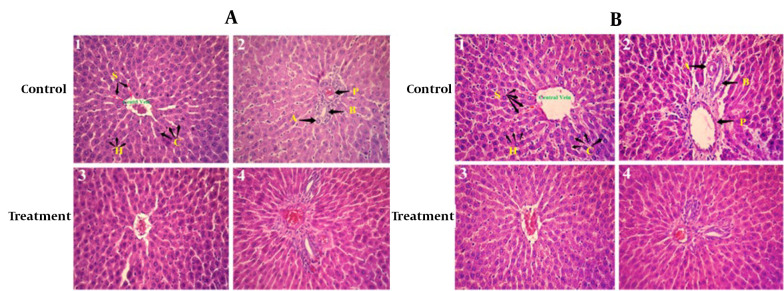
Photomicrograph of sectioned liver of A, female rats: Control and treated with 1 000 mg/kg Camelina oil; B, male rats: Control and treated with 1 000 mg/kg Camelina oil. A: Artery, P: Portal vein, B: Bile duct, H: Hepatocyte.

Histopathological examinations of the organs (heart, liver, and left kidney) of all rats exposed to sub-chronic treatment with Camelina oil showed no apparent lesions or structural alterations, even at the dose of 1 000 mg/kg on day 90 and magnification of ×400 (Figures 2 - 4). 

In the histology study of the left ventricular heart (Figure 2), the cardiac myocytes in the longitudinal section of the control group exhibited individual nuclei located in the center of the cell, with acidophilic cytoplasm and irregular skeletal muscle attachment around the cells. A similar normal appearance to the control group was observed in the longitudinal section of the left ventricular heart of the test rats administered 1 000 mg/kg Camelina oil.

The photomicrograph of the cortical section of the kidney of both treated and control groups (Figure 3) indicated that the glomerular capillaries, Bowman's capsule, afferent and efferent arterioles, and tubules did not show any structural alterations across all groups.

Liver histopathology of rats in both the control and treated groups, sub-chronically exposed to 1 000 mg/kg of Camelina oil (Figure 4), showed normal liver cells, a regular central vein, hepatic fibers, sinusoids, arteries, hepatic ducts, bile ducts, and portal veins. The liver section of the group treated with 1 000 mg/kg of Camelina oil exhibited a natural appearance similar to the control group.

## 5. Discussion

One of the first steps in safety evaluation is preclinical studies. These studies can provide valuable information regarding toxic effects and also help in calculating safe doses for conducting clinical studies. The main objective of this study was to determine the safety of dietary Camelina oil in acute and long-term periods (90 days) in Wistar rats.

In the acute toxicity study, no treatment-related behavioral changes, mortality, or other observable signs of toxicity following the administration of Camelina oil were observed. This indicates that the oral LD50 of the oil was considered to be greater than 5 000 mg/kg; therefore, the tested extracts can be categorized as relatively safe.

No significant alteration in the daily body weight of Wistar rats exposed to all doses of Camelina oil and 500 mg/kg body weight of Canola oil relative to the control over a long-term sub-chronic study was observed. In the organ/body weight ratio of vital organs, only the liver and left kidney showed a significant increase at the dose of 500 mg/kg in the male rats treated with Camelina oil compared to the control group. The increase in body weight of rats given Camelina sativa oil compared to the control group was favorable.

Several research studies conducted on dairy cows (8, 18-21) showed no effects of Camelina sativa oil seed diet on health or survival among treatment groups. Moriel et al. (22) showed that Camelina seed had no impact on the health performance and daily weight gain in dairy heifers. Moreover, the study by Frame et al. (23) showed that when Camelina oil seed was fed at a dietary level of 10% to Turkey Hens, there was no significant difference in weight gain compared to the control group.

In addition, Betankor et al. (24, 25) demonstrated that Camelina sativa oil and its transgenic form were used to replace fish oil in salmon feeds, and thus did not negatively affect fish health and growth performance. Similar results were obtained by Peiretti et al. (26) who supplemented the feed with three levels (0%, 10%, or 15%) of Camelina seed. These authors reported that the addition of Camelina seed to the diet of fattening rabbits at 15% for 50 days did not result in differences among the control groups in liver weight and body weight gain. Another 90 day study on broiler birds also reported (27) that the addition of 10% dietary Camelina seed to the broiler diet did not result in weight gain up to 6 weeks of age. Moreover, the relative weight of the liver and heart/body weight was not significantly altered with the addition of Camelina oil seeds to the diet.

Determination of hematological parameters is important in assessing physiological and pathological status. In the sub-chronic study for 90 days, no significant alterations were observed in any of the hematological parameters of the treated rats at all doses used when compared with the control group. Only the significant increase in WBC count in female rats (250 mg/kg) could be associated with the body's immune system. However, since doses above 250 mg/kg of Camelina did not show such an effect, the alteration does not seem to be related to Camelina administration.

The liver is the primary organ involved in drug metabolism. Levels of biochemical parameters of liver function such as AST and ALT usually determine the degree of liver damage or any toxicity effects (28). Moreover, an abnormally elevated ALP level in the blood indicates cholestatic diseases such as gallstones or tumors blocking the bile duct (29).

In the present study, there were no significant increases in plasma ALT and AST levels in female rats administered Camelina and Canola oils relative to the control, while the decrease in specific liver enzyme activity in male rats is not associated with liver toxicity (30). The edible oils also did not cause any significant increase in ALP activity in rats of both sexes. Plasma levels of total proteins like Albumin can be used as a criterion for evaluating the detoxification capacity of the liver, as almost all of them are produced by hepatocytes (31). Decreased total protein concentration can also be caused by chronic and acute diarrhea and liver disease.

In the present study, no changes or alterations in albumin concentration were recorded in female rats, whereas albumin levels in male rats treated with Camelina oil at a dose of 1 000 mg/kg decreased compared to the control group. It has been reported that an increase in globulin levels and a decrease in albumin levels result in a decrease in the albumin/globulin ratio, which is observed following damage to the integrity or function of liver cells (32, 33). It must be noted that microscopic studies demonstrated normal hepatocytes without lesions. Consequently, we concluded that this change was a normal physiological fluctuation in animals and had no toxicological significance.

Additionally, increases in phosphorus levels and decreases in calcium levels were found in male rats at all three tested doses. The common cause of hyperphosphatemia is renal insufficiency, leading to the inhibition of calcium efflux from the bone. Also, primary impairment in calcitriol synthesis due to early-stage renal failure contributes to hypocalcemia (34). It must be noted that these changes were also observed in the group that received Canola oil, which has been identified as a safe edible oil. Moreover, serum urea and creatinine concentrations indicate the likelihood of renal problems or dysfunction. The insignificant changes in these parameters alongside histopathologic experiments could suggest that renal function is not affected by Camelina oil.However, we cannot overlook their association with Camelina oil administration, and this matter requires further investigation.

Histopathology findings revealed that treatment of Wistar rats with Camelina oil at 1 000 mg/kg did not show significant microscopic changes in the normal architecture of the liver, left kidney, and heart. Our findings are in line with earlier studies (25, 35, 36). Ni Eidhin et al. (37) showed that Camelina oil, with a daily intake of about 8.9 or 10.2 g/kg BW/day, did not cause any adverse effects in 13-week-administered male/female swine. Rajapakse (38) also did not find any detrimental effects in broiler chickens by incorporating 7.7% Camelina oil in the diet meal.

More recently, a toxicological investigation of Camelina oil performed on adult dogs by Burron et al. (36) concluded that Camelina oil induced minimal changes in measured parameters compared to those treated with Canola oil, indicating its safety for use in the nutrition of adult dogs. Regarding human studies on Camelina oil, Karvonen et al. (39) carried out a controlled trial of Camelina oil supplementation (30 g/day) in the daily intake of Finnish adults. The results demonstrated lipid lowering along with no adverse effects of Camelina oil. Going one step further, Health Canada and European countries have already registered Camelina oil as a food-grade oil (40).

### 5.1. Conclusions

As discussed earlier, this is the first report presenting essential information about the effects of dietary Camelina oil supplementation in Wistar rats and the establishment of its safe dose for human nutrition. The Camelina seed oil cultured under Iranian conditions, orally administered to test Wistar rats, was relatively safe with an LD50 above 5 000 mg/kg BW compared to canola, supporting its classification at least in the fourth class of the world chemical classification system. Moreover, no animal mortality or specific changes in hematological and biochemical parameters were observed; significant hepatocellular, glomerular, or myocardial damages were not evident, suggesting that Camelina oil should be considered safe for human use.

## Data Availability

The dataset presented in the study is available on request from the corresponding author during submission or after publication.

## References

[1] Lydia DE, Mohandas A, Priya S, Monica SJ, Gajdacs M, Khusro A (2022). Physicochemical properties, nutrient profile, microbial stability, and sensory evaluation of cupcakes enriched with pomegranate seed oil.. Cell Mol Biol (Noisy-le-grand)..

[2] Omidi H, Tahmasebi Z, Naghdi Badi HA, Torabi H, Miransari M (2010). Fatty acid composition of canola (Brassica napus L.), as affected by agronomical, genotypic and environmental parameters.. C R Biol..

[3] Gil A, Serra-Majem L, Calder PC, Uauy R (2012). Systematic reviews of the role of omega-3 fatty acids in the prevention and treatment of disease.. Br J Nutr..

[4] Janssen CI, Kiliaan AJ (2014). Long-chain polyunsaturated fatty acids (LCPUFA) from genesis to senescence: the influence of LCPUFA on neural development, aging, and neurodegeneration.. Prog Lipid Res..

[5] Walker CG, Jebb SA, Calder PC (2013). Stearidonic acid as a supplemental source of omega-3 polyunsaturated fatty acids to enhance status for improved human health.. Nutr..

[6] Dawood MA (2020). Nutritional immunity of fish intestines: important insights for sustainable aquaculture.. Rev Aquac..

[7] Sprague M, Betancor MB, Tocher DR (2017). Microbial and genetically engineered oils as replacements for fish oil in aquaculture feeds.. Biotechnol Lett..

[8] Tejera N, Vauzour D, Betancor MB, Sayanova O, Usher S, Cochard M (2016). A Transgenic Camelina sativa Seed Oil Effectively Replaces Fish Oil as a Dietary Source of Eicosapentaenoic Acid in Mice.. J Nutr..

[9] Ghobadi R, Rostami Ahmadvandi H, Zeinodini A, Akbarabadi A (2021). Nutritional properties and benefits of camelina oil and meal.. Agrotech Ind Crops..

[10] Teimoori N, Ghobadi M, Kahrizi D (2023). Improving the growth characteristics and grain production of Camelina (Camelina sativa L.) under salinity stress by silicon foliar application.. Agrotech Ind Crops..

[11] Imbrea F, Jurcoane S, Halmajan HV, Duda M, Botos L (2011). Camelina sativa: A new source of vegetal oils.. Rom Biotechnol Lett..

[12] Zealand FSAN (2003). Erucic acid in food: A toxicological review and risk assessment.

[13] Tripathi MK, Mishra AS (2007). Glucosinolates in animal nutrition: A review.. Anim Feed Sci Technol..

[14] Fallah F, Kahrizi D, Rezaeizad A, Zebarzadi A, Zarei L (2020). [Evaluation of Genetic Variation and Parameters of Fatty Acid Profile in Doubled Haploid Lines of Camelina sativa L.].. J Plant Genetic Researches..

[15] Zubr J, Matthäus B (2002). Effects of growth conditions on fatty acids and tocopherols in Camelina sativa oil.. Ind Crops Prod..

[16] Raczyk M, Popis E, Kruszewski B, Ratusz K, Rudzińska M (2015). Physicochemical quality and oxidative stability of linseed (Linum usitatissimum) and camelina (Camelina sativa) cold‐pressed oils from retail outlets.. Eur J Lipid Sci Technologl..

[17] Rodríguez-Rodríguez MF, Sánchez-García A, Salas JJ, Garcés R, Martínez-Force E (2013). Characterization of the morphological changes and fatty acid profile of developing Camelina sativa seeds.. Ind Crops Prod..

[18] Hurtaud C, Peyraud JL (2007). Effects of feeding camelina (seeds or meal) on milk fatty acid composition and butter spreadability.. J Dairy Sci..

[19] Halmemies-Beauchet-Filleau A, Kokkonen T, Lampi AM, Toivonen V, Shingfield KJ, Vanhatalo A (2011). Effect of plant oils and camelina expeller on milk fatty acid composition in lactating cows fed diets based on red clover silage.. J Dairy Sci..

[20] Bayat AR, Kairenius P, Stefanski T, Leskinen H, Comtet-Marre S, Forano E (2015). Effect of camelina oil or live yeasts (Saccharomyces cerevisiae) on ruminal methane production, rumen fermentation, and milk fatty acid composition in lactating cows fed grass silage diets.. J Dairy Sci..

[21] Salin S, Taponen J, Elo K, Simpura I, Vanhatalo A, Boston R (2012). Effects of abomasal infusion of tallow or camelina oil on responses to glucose and insulin in dairy cows during late pregnancy.. J Dairy Sci..

[22] Moriel P, Nayigihugu V, Cappellozza BI, Goncalves EP, Krall JM, Foulke T (2011). Camelina meal and crude glycerin as feed supplements for developing replacement beef heifers.. J Anim Sci..

[23] Frame D, Palmer M, Ward R, Martini S (2008). Feeding Camelina sativa and enhancing omega-3 fatty acid levels in market-age turkey hens.. J Appl Poult Res..

[24] Betancor MB, Sprague M, Sayanova O, Usher S, Metochis C, Campbell PJ (2016). Nutritional Evaluation of an EPA-DHA Oil from Transgenic Camelina sativa in Feeds for Post-Smolt Atlantic Salmon (Salmo salar L.).. PLoS One..

[25] Betancor MB, Li K, Sprague M, Bardal T, Sayanova O, Usher S (2017). An oil containing EPA and DHA from transgenic Camelina sativa to replace marine fish oil in feeds for Atlantic salmon (Salmo salar L.): Effects on intestinal transcriptome, histology, tissue fatty acid profiles and plasma biochemistry.. PLoS One..

[26] Peiretti PG, Mussa PP, Prola L, Meineri G (2007). Use of different levels of false flax (Camelina sativa L.) seed in diets for fattening rabbits.. Livest Sci..

[27] Aziza AE, Quezada N, Cherian G (2010). Antioxidative effect of dietary Camelina meal in fresh, stored, or cooked broiler chicken meat.. Poult Sci..

[28] Alkali YI, Jimoh AO, Muhammad U (2018). Acute and Sub-chronic Toxicity Studies of Methanol Leaf Extract of Cassia singueana F. (Fresen) in Wistar Rats.. Herbal Med Open Access..

[29] Corathers SD (2006). Focus on diagnosis: the alkaline phosphatase level: nuances of a familiar test.. Pediatr Rev..

[30] Ozer J, Ratner M, Shaw M, Bailey W, Schomaker S (2008). The current state of serum biomarkers of hepatotoxicity.. Toxicol..

[31] Rasekh HR, Nazari P, Kamli-Nejad M, Hosseinzadeh L (2008). Acute and subchronic oral toxicity of Galega officinalis in rats.. J Ethnopharmacol..

[32] Valášková P, Muchová L (2016). Metabolism of bilirubin and its biological properties.. Klinická biochemie a metabolismus..

[33] Eric Y, Arooj B, Moaz C, Matthew K, Nikolaos P (2016). Acetaminophen-Induced Hepatotoxicity: a Comprehensive Update.. J Clin Transl Hepatol..

[34] Barreto FC, Barreto DV, Massy ZA, Drueke TB (2019). Strategies for Phosphate Control in Patients With CKD.. Kidney Int Rep..

[35] Pawlowska-Olszewska M, Puzio I, Harrison AP, Borkowski L, Tymicki G, Grabos D (2018). Supplementation with camelina oil prevents negative changes in the artery in orchidectomized rats.. J. Physiol. Pharmacol..

[36] Burron S, Richards T, Patterson K, Grant C, Akhtar N, Trevizan L (2021). Safety of Dietary Camelina Oil Supplementation in Healthy, Adult Dogs.. Animals (Basel)..

[37] Ni Eidhin D, Burke J, Lynch B, O'Beirne D (2006). Effects of Dietary Supplementation with Camelina Oil on Porcine Blood Lipids.. J Food Sci..

[38] Rajapakse B (2015). Nutritive evaluation of mechanically-pressed camelina (Camelina sativa), carinata (Brassica carinata) and soybean (Glycine max) meals for broiler chickens [Master thesis]..

[39] Karvonen HM, Aro A, Tapola NS, Salminen I, Uusitupa MI, Sarkkinen ES (2002). Effect of alpha-linolenic acid-rich Camelina sativa oil on serum fatty acid composition and serum lipids in hypercholesterolemic subjects.. Metab..

[40] Gupta SK (2016). Biology and Breeding of Crucifers..

